# Antioxidant Activities and Caffeic Acid Content in New Zealand Asparagus (*Asparagus officinalis*) Roots Extracts

**DOI:** 10.3390/antiox7040052

**Published:** 2018-04-04

**Authors:** Abbey Symes, Amin Shavandi, Hongxia Zhang, Isam A. Mohamed Ahmed, Fahad Y. Al-Juhaimi, Alaa El-Din Ahmed Bekhit

**Affiliations:** 1 Department of Food Science, University of Otago, Dunedin 9054, New Zealand; abbeysymes77@gmail.com (A.S.); amin.shavandi@otago.ac.nz (A.S.); hongxia.zhang@postgrad.otago.ac.nz (H.Z.); 2 Department of Food Science and Nutrition, College of Food and Agricultural Sciences, King Saud University, Riyadh 11451, Saudi Arabia; isamnawa@yahoo.com (I.A.M.A.); faljuhaimi@ksu.edu.sa (F.Y.A.-J.)

**Keywords:** asparagus roots, antioxidant activity, bioactive compounds

## Abstract

*Asparagus officinalis* are perennial plants that require re-planting every 10–20 years. The roots are traditionally mulched in the soil or treated as waste. The *A. officinalis* roots (AR) contain valuable bioactive compounds that may have some health benefiting properties. The aim of this study was to investigate the total polyphenol and flavonoid contents (TPC and TFC, respectively) and antioxidant (2,2-diphenyl-1-picrylhydrazyl (DPPH), Oxygen Radical Absorbance Capacity (ORAC) and Ferric Reducing/Antioxidant Power (FRAP) assays) activities of New Zealand AR extract. The antioxidant activity decreased with a longer extraction time.

## 1. Introduction

Asparagus belongs to the *Asparagaceae* genus, which originated in the Eastern Mediterranean region and Asia. Asparagus is a versatile plant with a unique texture and flavour that has promoted its consumption worldwide. There are 22 different species of asparagus recorded in India [1]. Most of the research on the bioactive properties of asparagus extracts was carried out on extracts obtained from the shoots of *Asparagus officinalis* and the roots of *A. racemosus* [2,3]. Different parts of *A. racemosus* have been suggested to have health benefits such as improving the immune system and cancer prevention [4,5]. *A. officinalis* is mainly cultivated for the consumption of the shoot, but its roots have no value and very little research has been carried out on bioactives in the roots of this important plant.

*A. officinalis* can be harvested for up to 40 years or until their productivity and quality decline. The quality of asparagus declines over time due to diseases from microorganisms, such as *Fusarium*, *Phytophthora*, *Stemphylium*, *Phomopsis asparagi*, and *Cercospora asparagi* Sacc species, as well as the autotoxins that asparagus itself produces, which hinder the growth of new asparagus plants [6]. Therefore, the regeneration of the rootstock becomes necessary for productivity. Old asparagus roots are considered as a waste in many countries, including New Zealand, and are commonly left to rot in fields. However, asparagus waste, not including the roots, has been reported as a potential source of bioactive compounds such as saponins, polyphenols, and flavonoids [7]. These bioactive compounds could be extracted from the roots, hence utilising the roots in a manageable manner and offers the opportunity to add value to the production process. The roots also possess allopathic properties, which can prevent other asparagus plants from growing. Therefore, farmers could improve the re-growth of new asparagus by removing the allopathic potential of old roots, as well as increasing their income by selling the roots, allowing the recipients of the bioactive compounds to have access to a natural product with potential health benefits. Environmental factors such as temperature and exposure to UV and soil nutrients can affect the synthesis and the content of bioactives in plants. Therefore, the objective of this study was to determine the antioxidant activity of New Zealand *A. officinalis* root extracts obtained using methanol and ethanol as the most commonly used extraction solvents. 

## 2. Materials and Methods

Methanol, ethanol, sodium carbonate, acetonitrile, trifluoroacetic acid, and sodium acetate were obtained from Fisher Scientific (Waltham, MA, USA). Gallic acid, sodium nitrite, rutin, 2,2-diphenyl-1-picrylhydrazyl (DPPH), fluorescein sodium salt, 6-hydroxy-2,5,7,8-tetramethylchroman-2-carboxylic acid (Trolox), 2,4,6-tris(2-pyridyl)-s-triazine (TPTZ), caffeic acid, saponin, and ferrous sulphate were obtained from Sigma-Aldrich (St. Louis, MO, USA). Folin-Ciocalteu reagent and di-sodium phosphate decahydrate were purchased from Merck Millipore Corporation (Billerica, MA, USA); aluminium chloride from Loba Chemie (Mumbai, India); sodium hydroxide and glacial acetic acid from VWR International, (Radnor, PA, USA), sodium dihydrogen orthophosphate 1-hydrate and ferric chloride from BDH Chemicals LTD (Poole, UK); 2,2′-azobis-2-methyl-propanimidamide dichloride (AAPH) from the Cayman Chemical Company (Ann Arbor, MI, USA). All chemicals used were of analytical grade.

### 2.1. Preparation of Asparagus Roots

New Zealand green and purple AR roots were obtained from a commercial farm in Palmerston (New Zealand) (40.3523° S, 175.6082° E) from plants that had been planted for 15 years. The *A. officinalis* roots (AR) were cleaned from soil and other contaminants were removed from the roots by thoroughly washing them with tap water and then rinsing them with deionized water [8]. Then the roots were dried using an oven (Contherm 2150, Contherm Scientific Limited, Upper Hut, New Zealand) at 60 °C for 72 h. The dried roots were then pulverized into a powder using a Jingangdikai JG100 grinder (Jingangdikai Co., Guandong, China) and sieved through a 40 mm mesh. The dried root powder samples were transferred into closed plastic containers and kept at −20 °C until further analysis.

### 2.2. Extraction of Bioactive Compounds

The effects of extraction solvent (water, methanol or ethanol), solvent concentration, number of extractions (reflect the number of extractions of the same sample) and extraction time on the yield and antioxidant activities of the extracts were investigated. A modified method of Fan et al. [9] was carried out for the extraction of bioactive compounds from the AR. The AR powder was extracted using deionized water, methanol or ethanol at different concentrations (50%, 70%, and 90%). The samples were extracted twice and all extractions were carried out with triplicate samples. For the first extraction, 10 g of the AR powder was combined with 200 mL of the extraction solvent in a flask. The samples were then wrapped in aluminium foil and placed into a shaking incubator (Ratek, Victoria, Australia) at 70 °C for 2 h shaking at a speed of 80 RPM. After 2 h, the samples were centrifuged (J2-21 M/E Beckman, Brea, CA, USA) at 10,000× *g* for 20 min and the supernatant was filtered using Whatman No 1 filter paper by vacuum. The methanol and ethanol in the extracted samples were removed using a Rotavapor^®^ (Büchi, Chadderton, UK) at 40 °C, and then the samples were frozen at −80 °C before being freeze-dried (Labconco, Kansas City, MO, USA). The pellets from the centrifuge and filter steps were subjected to a second extraction as described above. The samples from both extractions were kept separate and referred to in the following sections as “extraction order”. The samples were frozen at −80 °C and freeze-dried. This process was repeated using the same solvent: solid ratio, and extraction temperature using ethanol as the extraction solvent at a longer extraction time of 10 h. The yield was calculated using the following equation:

$$\mathrm{Yield}\;\left(\text{\%}\right)=\frac{\left(\mathrm{Weight}\;\mathrm{of}\;\mathrm{freeze}-\mathrm{dried}\;\mathrm{extract}\right)}{\mathrm{Weight}\;\mathrm{of}\;\mathrm{dried}\;\mathrm{asparagus}\;\mathrm{roots}}\;\times \;100$$

### 2.3. Analysis of Asparagus Root Extracts

The samples were analysed for their TPC (Total phenolic content), TFC (Total phenolic content) and antioxidant activities using solutions prepared fresh on the day of analysis (1 mg/mL in 50% methanol), kept on ice and protected from light. All analyses were carried out in triplicate measurements.

### 2.4. Total Phenolic Content

To determine the TPC of the samples, the Folin-Ciocalteu colourimetric method was carried out using a plate reader (Biotek, Winooski, VT, USA) as described by Farasat et al. [10]. In a 96-well microplate, 20 µL of the extract was added, followed by 100 µL of Folin-Ciocalteu reagent (1:10) and 80 µL of sodium carbonate (7.5%). The plate was left in the dark at room temperature for 30 min, and the absorbance was read at 600 nm. Gallic acid was used to construct a standard curve (0–0.15 mg/mL), hence the polyphenol content of the samples was expressed as Gallic Acid Equivalents per gram of extract (mg GAE/g extract).

### 2.5. Total Flavonoid Content

The aluminium chloride colourimetric method of Herald et al. [11] was carried out using a plate reader to determine the TFC. In a 96-well microplate, 100 µL of distilled water, 10 µL of sodium nitrite (50 g/L), and 25 µL of the extract solution were added to each well. After 5 min of incubation in the dark at room temperature, 15 µL of aluminium chloride (100 g/L) was added and the mixture was left for a further 6 min in the dark at room temperature. Finally, 50 µL of sodium hydroxide (1 mol/L) and 50 µL of distilled water were added to the wells. The absorbance of the mixture was measured at 510 nm and rutin was used to construct a standard curve (1–0.05 mg/mL), hence the flavonoid content of the samples was expressed as rutin equivalent per gram of extract (mg RE/g extract).

### 2.6. Antioxidant Activity

#### 2.6.1. Determination of DPPH Radical Scavenging Activity

The DPPH assay was conducted using the method described by De-Ancos et al. [12] using a plate reader. A 10 µL of the sample and 90 µL of deionized water were added to each well of a 96-well microplate. Then, in dimmed light, 100 µL of DPPH (78 mg/L) was added to each well. The plate was left in the dark at 25 °C for 30 min before the absorbance was measured at 517 nm. Gallic acid was used to construct a standard curve, and the DPPH scavenging activity was determined as DPPH inhibition (%) from the equation:

$$\mathrm{DPPH}\;\mathrm{Inhibition}=\frac{\left(1-\mathrm{test}\;\mathrm{sample}\right)}{\mathrm{blank}\;\mathrm{sample}\;\mathrm{absorbance}}\;\times 100$$

#### 2.6.2. Determination of Oxygen Radical Absorbance Capacity (ORAC)

The method of Huang et al. [13] was used to determine the ORAC. Phosphate buffer (75 mM, pH 7.4) was prepared and a stock solution of 1 mM of fluorescein sodium salt was made using the buffer, wrapped in aluminium foil and kept at 4 °C. This stock solution was diluted further to make a 10 nM solution, which was used on the day of the assay. The AAPH was made by dissolving 0.0646 g in 1 mL of the phosphate buffer. The 10 nM fluorescein and AAPH solutions were kept on ice in the dark until use and were made fresh daily. For the standard curve Trolox (0–150 mM) was prepared in phosphate buffer and used to construct the standard curve.

A 25 μL of the standard or sample and 150 μL of the 10 nM fluorescein were pipetted into a 96-well microplate in the dark. Then the plate was covered in parafilm and placed in the plate reader at 37 °C for 30 min. A 25 μL of APPH was added, the plate was shaken for 20 s, and the fluorescence was read at excitation at 485 nm and emission at 527 nm, at 1-min intervals over 1.5 h. The results were analysed by calculating the AUC, using the following equation:

$$\mathrm{AUC}=1+\;\overset{t_{i}=90\;\min}{\underset{t_{0}=0\;\min}{\sum }}\frac{A_{i}}{A_{0}}$$

where *A*_0_ is the initial fluorescence reading at *t*_0_ and *A_i_* is the reading at *t_i_* hence, the final ORAC results were the difference between the area under the curve the blank and the samples. The results were expressed as mM Trolox equivalents per gram of extract (mM TE/g extract).

#### 2.6.3. Ferric Reducing Ability Power (FRAP) Assay

The method of Benzie and Strain [14] was used with some modifications as reported by Teh et al. [15] so that it could be applied to a plate reader in a 96-well microplate. A FRAP solution was made in acetate buffer (300 mM, pH 3.6) that contained 10 mM TPTZ, 20 mM FeCl_3_ and deionized water. The 10 mM TPTZ was made up in 10 mL of HCl (40 nM) and placed in a water bath at 50 °C until the compound was dissolved. The 20 mM FeCl_3_ was made up in 10 mL of distilled water.

FRAP solution 100 mL of the acetate buffer (300 mM, pH 3.6), 10 mL of TPTZ (10 mM) and 10 mL of FeCl_3_ were combined and kept at 37 °C until use. This solution was made fresh daily. For the standard curve, iron sulphate was used (0–1.5 mM). In a 96-well microplate, 10 µL of the standard (iron sulphate) or sample was added, followed by 200 µL of the FRAP solution. The plate was incubated at 37 °C for 4 min inside the plate reader. The results were expressed as mM iron sulphate equivalents per gram of extract (mM FeSO_4_ E/g extract).

### 2.7. HPLC Analysis

Reverse phase HPLC [9] was used to separate and quantify the bioactive compounds extracted from the different extraction methods. All of the samples were filtered into 2 mL amber vials using a 0.45 μm nylon filter (Phenomenex, Torrance, CA, USA). The amber vials were then placed into the auto-sampler chamber, which was kept at 4 °C during the analysis. The HPLC used was an Agilent 1200 (Agilent Technologies, Santa Clara, CA, USA), with autosampler and Diode Array Detector (DAD). The column used was a Luna C18 (5 μm, 250 × 4.6 mm, Phenomenex, Torrance, CA, USA), with a 10 µm 4 × 3 mm C18 guard column (Phenomenex, Torrance, CA, USA). The column was maintained at 25 °C. The mobile phase consisted of Milli-Q water (A) and acetonitrile containing 0.02% *v*/*v* of trifluoroacetic acid (TFA) (B). Preliminary work using several standards (quercetin, rutin, gallic acid, ferulic acid, and caffeic acid) showed that the main compound in AR is caffeic acid. The caffeic acid content in the obtained extracts of the analytes was identified and quantified by comparing the retention time of the peak with a reference standard eluted under the same conditions.

### 2.8. Statistical Analysis

The statistical analysis was carried out using Minitab 16 Statistical Software (State College, PA, USA) using the general linear model (GLM) protocol. The investigated parameters were the TPC, TFC, caffeic acid and DPPH, ORAC and FRAP activities of AR extracts. For the solvent extraction method, a multivariate analysis of variance (ANOVA) was carried out to examine the effects of extraction solvent (methanol, ethanol, and water), solvent concentration (50%, 70%, and 90%), extraction order (1st and 2nd extraction) and their interactions on the measured parameters. The results for 2-h and 10-h extraction times using ethanol were analysed separately. Tukey’s honestly significant difference (HSD) post hoc test was used for multi comparison tests. A Pearson’s correlation was also carried out to determine the correlations among the measured parameters.

## 3. Results and Discussion

In agreement with the literature, the conditions required for the optimal conventional extraction method of bioactive compounds from plant materials depended greatly on the extraction order and time, extraction solvent and concentration [16]. 

### 3.1. Extraction Time and Order on the Yield

There was a significant effect for the extraction order (*p* < 0.05) in both 2-h and 10-h extractions, where the 1st extraction had a significantly higher yield compared to the 2nd extraction (Table 1). A careful evaluation of the economics of the process needs to be considered as to whether it would be worthwhile for a 2nd extraction to be carried out (i.e., the yield and activity would have to provide enough incentive for the extra processing time and cost). The yield from 10-h 1st extraction was significantly higher than the 2-h comparable extraction (*p* < 0.01, data not shown). The obtained extracts reflected a mix of several cellular biomaterials, including carbohydrates. Therefore, it was important to evaluate the bioactivity of the extract rather than limiting the evaluation on crude extract, since the maximum amount of bioactive compounds extracted by the solvent may have varied due to differences in the compounds’ affinity toward the various extraction solvents.

### 3.2. Effect of Extraction Solvent on TPC, TFC, and Antioxidant Activity

There were significant effects for the extraction solvents during 2-h extraction time on the TPC, TFC and FRAP activity (*p* < 0.001) but not the DPPH scavenging (DPPH) and ORAC activities (Table 2 and Table 3). There were no differences between ethanol and methanol for TFC and FRAP activity. Given that methanol led to a small increase (about 5–7%) in TPC and with the known toxicity of methanol, it was decided that ethanol was the best extraction solvent as it would be more acceptable as an extraction solvent for health reasons [17,18,19]. The best extraction solvent to extract the maximum amount of bioactive compounds is dependent on the type of plant matrix in question [16]. Different amounts of antioxidant compounds were extracted by methanol, ethanol and water from plants materials, which was depended on the polarity, density and pH [16]. Hence, different antioxidant compounds have varying optimal extraction conditions. Although polyphenols and flavonoids are polar compounds, they express maximum extraction potential when are extracted from a solvent containing a combination of polar and non-polar substances [20]. Therefore, it was possible that different compounds were extracted in the different extraction solvents. The best extraction solvent determined in this research is in agreement with the literature on the extraction of bioactives from asparagus, as ethanol was found to be the most popular extraction solvent [2,3,9,21,22]. Methanol was found to be the optimum extraction solvent when it was used as pure methanol in a Soxhlet system [23,24]. During preliminary trials, a Soxhlet extraction method was carried out; however, it was found to be time-consuming and required a high amount of solvent, which resulted in high cost and a lot of organic waste to manage. Ideally, the best method needed for this type of research should be simple and easy to carry out, so that the commercialization pathway is affordable and commercially viable. Therefore, the ethanol extraction process was chosen for further investigations.

**Figure 1:**
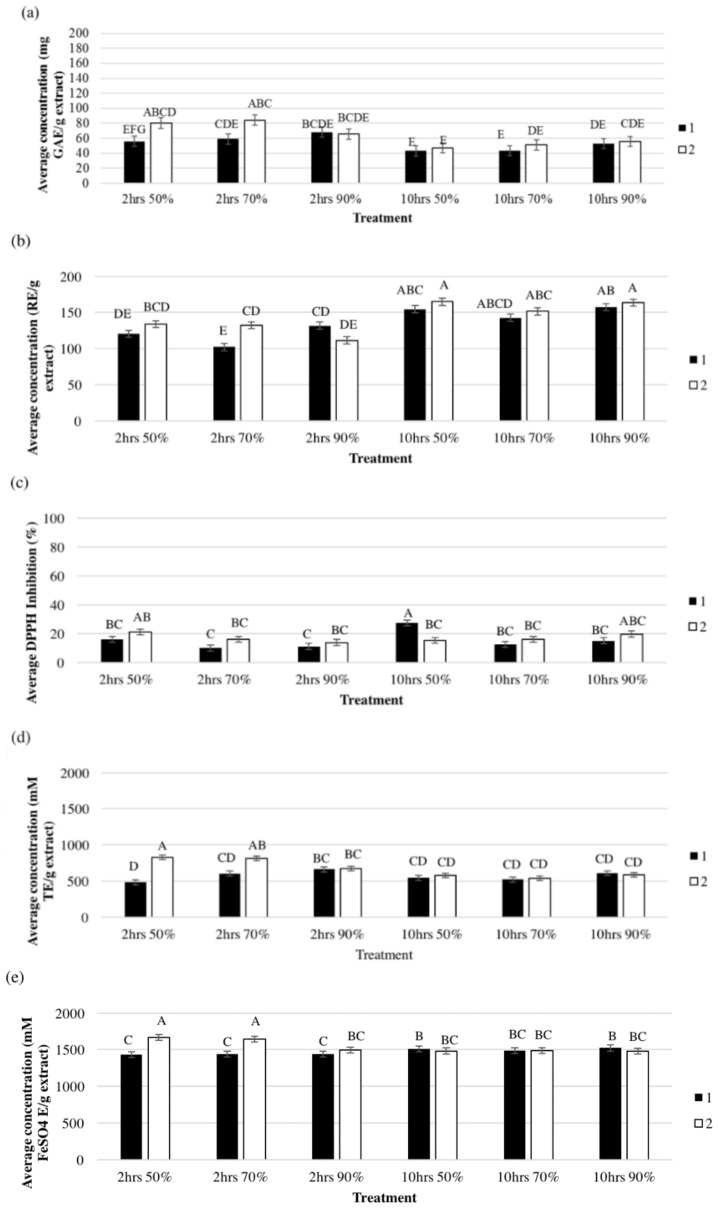
The effect of ethanol concentration, extraction order and extraction time on total phenolic content (**a**); total flavonoid content (**b**); 2,2-diphenyl-1-picrylhydrazyl (DPPH) scavenging activity (**c**); Oxygen Radical Absorbance Capacity (ORAC) activity (**d**); and Ferric Reducing/Antioxidant Power (FRAP) activity (**e**) of the extract obtained from asparagus root powder after 2 and 10-h extraction time. Bars that do not share the same letter are significantly different (*p* < 0.05).

The extraction was carried out using two extractions times; 2-h and 10-h. The 2-h and 10-h extraction times resulted in different (*p* < 0.05) antioxidant activities (Figure 1). For the TPC, ORAC, and FRAP, a 2-h extraction had higher activities compared to 10-h extraction. For the TFC and DPPH activity, the opposite was true. Fan et al. [9] investigated a range of extraction times up to 2.5 h and concluded that an extraction time of 2-h was sufficient to extract the maximum amount of antioxidant compounds from asparagus residue. From time 0 to 2.5 h, it was found that the amount of antioxidants extracted increased until it reached a constant value at 2 h and then decreased slightly with the increase in extraction time. This was not found in this research, as the amount of antioxidants extracted from the 10-h extraction was not less than the 2-h extraction. Solana, Boschiero, Dall’Acqua, and Bertucco [24] determined that in whole *A. officinalis* and found a longer extraction time (4 h) allowed more phenolic acids to be extracted.

In the present study, a long extraction time resulted in a lower amount of total polyphenols (Figure 1a). This was in agreement with Shi et al. [25] findings in grape seeds. Oxidation of the polyphenols during the long processing time may have resulted in polymerised insoluble compounds and reduced total polyphenols [25]. 

There was a significant effect for the extraction order (*p* < 0.05) in both 2-h and 10-h extractions, where the 1st extraction had a significantly higher yield compared to the 2nd extraction. The yield from the 10-h 1st extraction was significantly higher (*p* < 0.05) than the 2-h comparable extraction. The use of a 2nd extraction step appears to be important as it led to up to 20% recovery of yield, such as with 90% ethanol (Table 1). Other materials in the literature determined that a two-step extraction process resulted in the maximum amount of antioxidant compounds [25,26,27]. 

In the 2-h extraction, TPC per gram extract from the 1st and 2nd extractions were not different and a similar trend was found with TFC. However, ORAC and FRAP activities were affected (*p* < 0.05) by the extraction order (Figure 1). For the 10-h extraction, higher TPC and TFC (*p* < 0.05) were found in extracts from the 2nd extraction compared to the 1st extraction (Figure 1). Nawaz, Shi, Mittal, and Kakuda [27] investigated the optimal number of extractions to recover the maximum amount of polyphenols from grape seeds. The authors found that two extractions were ideal for that purpose. They found that the diffusion rate of the solute from the grape seeds decreased with increasing number of extractions. The extraction of bioactive is regulated by the diffusion of the extraction solvent into the matrix particles, where the driving force is the difference in pressure; and also by the diffusion of the antioxidant compounds from the matrix into the extraction solvent, where the driving force is the concentration difference [27]. As more antioxidant compounds were removed in the 1st extraction step, the concentration difference would have decreased, resulting in less compounds being extracted. Additional antioxidant compounds were extracted in the 2nd extraction due to disruption of the matrix during the 1st extraction, allowing the solvent in the 2nd extraction to diffuse more easily and extract more bioactives [28]. Hence, several researchers found a 3rd extraction is not justified (Nawaz, Shi, Mittal and Kakuda [27]; Cacace and Mazza [29]. In terms of yield, a 2nd extraction step would require economical evaluation in terms of benefit-cost relationship.

### 3.3. Effect of Extraction Time and Order on the Antioxidant Compounds

The optimal condition to extract TPC involved a 2-h extraction and the use of 70% ethanol. In all cases, the 2nd extraction had slightly more, but insignificant TPC compared to the 1st extraction, except for 2-h 50% ethanol (Figure 1a). For TFC, the 10-h extraction had more TFC compared with the 2-h extraction. The optimal conditions for TFC extraction were 10-h extraction using 50% ethanol. Similar to the TPC, the 2nd extraction had slightly more, but insignificant TFC compared to the 1st extraction, with the exception of 2-h 70% ethanol treatment.

The optimal conditions for the extraction of DPPH inhibition activity were using a 10-h extraction with 50% ethanol. Under these conditions, the extract from the 1st extraction significantly (*p* < 0.05) had more antioxidant activity compared to the 2nd extraction. The optimal conditions for the extraction of ORAC antioxidant activity were 2-h extraction using 70% ethanol, which had significantly (*p* < 0.05) higher ORAC activity compared to the 10-h extraction. Even though the 2-h and 50% ethanol had the highest (*p* < 0.05) ORAC antioxidant activity in the 2nd extraction, the 2-h and 70% ethanol treatment resulted in a combined higher ORAC activity.

The 2-h 50% and 70% ethanol treatments had significantly (*p* < 0.05) higher FRAP antioxidant activity compared to the other treatments. The optimal conditions for extracts for that activity were 2-h at 50% ethanol. The 2nd extraction had more FRAP antioxidant activity compared to the 1st extraction in most cases (Figure 1e). This could be due to the matrix of the AR, as the 1st extraction would have disrupted the matrix, allowing the 2nd extraction to easily remove more antioxidants [27]. Furthermore, the removal of carbohydrates and other soluble compounds during the first extraction can help more specific extraction of antioxidants. To determine the optimal conditions to extract antioxidant compounds from AR, the extraction yields were calculated and are shown in Table 1. There were no effects (*p* > 0.05) for the solvent or the solvent concentration on the yield of AR extracts (Table 1).

### 3.4. Effect of Extraction Order and Solvent Type on Antioxidant Activity

The interaction between extraction order and extraction solvent investigated for the 2-h extraction, as the 10-h extraction only examined the use of one solvent “ethanol”. The extraction order and extraction solvent and their interaction had significant (*p* < 0.05) effects on the antioxidant activities of the extracts, which was dependent on the antioxidant assay used (Table 2 and Table 3). The 1st extraction did not have a significant effect (*p* > 0.05) on DPPH inhibition activity compared to the 2nd extraction for all the solvents used. For the ORAC activity, only the 50% and 70% 2nd extraction of ethanol were significantly higher (*p* < 0.05) than the other extractions. Ethanol 90% and water 1st extracts had significantly (*p* < 0.05) lower FRAP activity than their 2nd counterpart extracts. The 2nd methanol (50%, 70%, and 90%) and ethanol (50% and 70%) extracts had significantly (*p* < 0.05) higher FRAP activity found compared to water and ethanol 90% extracts. In general, methanol and ethanol extracts were similar in their antioxidant activity, while water extracts were significantly (*p* < 0.05) lower in most cases.

### 3.5. Effect of Solvent Concentration

Methanol and ethanol at different concentrations extracted the same amounts of TFC that were not different from water (Table 2). It is worth mentioning that the differences between ethanol and methanol in terms of TFC were less than 5–6%, which economically may not be attractive to justify the use of a toxic chemical such as methanol. Methanol 70% extract had the highest DPPH radical scavenging and ORAC activity (Figure 1 and Table 2). The FRAP activity of water and 90% ethanol extracts were lower than the other treatment with the exception of 50% methanol extract (Figure 1). Generally, 70% methanol and 50% ethanol were good extraction solvents for antioxidants of AR, and water was the least effective extraction solvent. In the 10-h extraction method, only ethanol was used since generally little differences were observed between ethanol and methanol in 2-h extraction. The optimal solvent concentration for the extraction of antioxidant compounds from plants depends on the matrix of the plant and the properties of the antioxidant compounds [16]. Optimal ethanol concentration used for the extraction of bioactives from different asparagus types and parts ranged from 50% to 80% [2,3,9,21,22], which is in agreement with the findings in this study. This wide range of concentrations could be due to the polarity of the antioxidant compounds, and the affinity they have towards the extraction solvent [16]. Different solvent concentrations may be able to diffuse in the AR structure and extract different types and amounts of antioxidant compounds. Shi, Yu, Pohorly, Young, Bryan, and Wu [25] and Fan, Yuan, Wang, Gao, and Huang [9] reported that a concentration of 50% ethanol was optimal as it allowed the maximum amount of the water-soluble polyphenols to be extracted. Furthermore, 50% ethanol has been known to be effective at extracting flavonoids [30], which is in accordance with the findings of this study.

### 3.6. Effect of Extraction Order and Solvent Concentration on the Antioxidant Activity

Only FRAP activity in 2-h extracts was significantly (*p* < 0.05) affected by the extraction order and the 2nd extraction had higher FRAP activity compared to the 1st extraction in all the solvents. In the 10-h extraction, TPC and DPPH activity were affected by the extraction order and solvent concentration (*p* < 0.05). Extracts obtained from the 90% solvent concentration significantly had higher TPC compared to 50% and 70% (Figure 1). There was an effect for the extraction order on TPC which was observed in 70% solvent concentration only. For the DPPH, 50% ethanol the first extraction had the highest DPPH scavenging activity (Figure 1). This could have been due to the antioxidants responsible for the inhibition of DPPH being more favourable to be extracted by 50% ethanol due to the polarity [16]. The concentration did not greatly influence the extraction of antioxidant compounds, apart from DPPH. This is in agreement with the findings of Diankov et al. [31] who found a negligible concentration effect when extracting antioxidant compounds in lemon peels.

### 3.7. Pearson Correlations for the Measured Activities in AR Extract

Pearson correlations analysis was carried out on the 2-h extraction and the 10-h extraction to examine the influence of extraction time on these relationships (Table 4). In the 2-h extraction, there were many positive correlations (*p* < 0.05). The DPPH assay and flavonoids had a positive correlation. This meant that the TFC were affecting DPPH inhibition activity. The FRAP and ORAC assays both had positive correlations to polyphenols and flavonoids, therefore the polyphenols and flavonoids contributed towards the FRAP and ORAC antioxidant activities. The FRAP and ORAC assays also had a positive correlation with each other. This meant that these two methods may have targeted the same type of antioxidant. The positive correlation between antioxidants was in agreement with previous findings for basil leaves [16] and spinach [32]. 

### 3.8. Analysis of AR Extract Using HPLC

Caffeic acid is a hydrocinnamic acid, and it contributes towards the autotoxicity in *A. officinalis* roots [33]. The major phenolic peak detected in *A. officinalis* roots is controversial with rutin [34] or caffeic acid [33,35] being alternatively reported as the highest phenolic compound in the roots of *A. officinalis* L. Although literature reported the presence of rutin in *A. officinalis* this was not reflected in the results of this study, as rutin was not found. This could have been due to the preparation conditions of *A. officinalis* AR samples, as Makris and Rossiter [36] found rutin degraded when *A. officinalis* spears were chopped. As shown in Table 2, after 2-h extraction the amount of caffeic acid was not affected by the solvent type (*p* > 0.05). Ethanol concentration and extraction order affected the caffeic acid content (Table 2), where 90% ethanol extract had higher caffeic acid content than the 50% and 70% ethanol extracts in the 1st extraction. Similarly, using 90% ethanol, 2.4 (mg/g extract) of caffeic acid was obtained after 10 h that was significantly higher than the 1.5 and 1.3 mg/g extract obtained with 50% and 70% ethanol, respectively. Studies have shown that caffeic acid and its derivatives exhibit significant biological activities such as antioxidants to control lipid peroxidation [37] and have a potential therapeutic effect in treating neurodegenerative diseases [38].

## 4. Conclusions

The antioxidant activities of green asparagus root were determined using conventional hydro-alcohol extraction methods. It was concluded that one extraction was sufficient to extract the majority of antioxidant compounds as a 2nd extraction had a significantly lower yield and so would not have been worthwhile, in respect to the time and extraction solvent required. The optimal conditions were found to be using 50% ethanol for 2-h, with a yield of 44%. The 2-h extraction time allowed more TPC, ORAC, and FRAP activities to be obtained. Although more flavonoids were found in the 10-h extraction, they did not appear to contribute more towards these antioxidant activities. Rutin was not found in New Zealand *A. officinalis* roots and caffeic acid was found to be the dominant phenolic in AR extracts. The extraction method developed in this study was simple and easy to carry out. Therefore, this method would enable the use of asparagus roots as a valuable-by product and hence prevent them from being a waste. 

## Figures and Tables

**Table 1:** The yield of extracts (Mean ± SEM) obtained using solvent extraction methods. ^a,b^ Within each extraction and ^x–z^ within each extraction time, values that do not share the same letter are significantly different (*p* < 0.05).

Extraction Time	Extraction Solvent	Extraction Order	Average Yield (%)
2-h	50% Methanol	1	42.4 ± 1.8 ^a^
	70% Methanol	1	42.1 ± 1.8 ^a^
	90% Methanol	1	41.8 ± 1.8 ^a^
	50% Ethanol	1	44.0 ± 1.8 ^a^
	70% Ethanol	1	44.3 ± 1.8 ^a^
	90% Ethanol	1	36.4 ± 1.8 ^a^
	Water	1	38.4 ± 1.8 ^a^
	50% Methanol	2	3.2 ± 1.8 ^b^
	70% Methanol	2	3.4 ± 1.8 ^b^
	90% Methanol	2	3.0 ± 1.8 ^b^
	50% Ethanol	2	3.6 ± 1.8 ^b^
	70% Ethanol	2	5.1 ± 1.8 ^b^
	90% Ethanol	2	7.0 ± 1.8 ^b^
	Water	2	4.2 ± 1.8 ^b^
10-h	50% Ethanol	1	53.6 ± 3.4 ^x^
	70% Ethanol	1	60.1 ± 3.4 ^x^
	90% Ethanol	1	29.8 ± 3.4 ^y^
	50% Ethanol	2	12.8 ± 3.4 ^z^
	70% Ethanol	2	6.4 ± 3.4 ^z^
	90% Ethanol	2	7.2 ± 3.4 ^z^

**Table 2:** Summary of antioxidant activities of asparagus root extract obtained from 2-h extraction. ^a–h^ Values that do not share the same letter are significantly different (*p* < 0.05).

Solvent	Total Polyphenols (mg GAE/g Extract)		Total Flavonoids (mg RE/g Extract)		DPPH Inhibition %		ORAC Antioxidant Activity (mM TE/g Extract)		FRAP Antioxidant Activity (mM FeSO_4_ E/g Extract)		Caffeic Acid (mg/g Extract)	
	Order		Order		Order		Order		Order		Order	
	1	2	1	2	1	2	1	2	1	2	1	2
Methanol 50%	61.5 ^efg^	78.8 ^efg^	128.6 ^d^	126.6 ^d^	23.6 ^d^	40.8 ^abcd^	557.6 ^de^	596.9 ^ade^	1445.0 ^d^	1572.5 ^bc^	2.5 ^ab^	2.7 ^ab^
Methanol 70%	67.9 ^efg^	84.7 ^defg^	122.5 ^de^	127.7 ^d^	47.0 ^a^	42.3 ^abcd^	776.1 ^abc^	676.5 ^abcd^	1439.8 ^d^	1632.1 ^ab^	2.2 ^ab^	2.7 ^ab^
Methanol 90%	64.9 ^efg^	91.4 ^def^	118.0 ^de^	123.3 ^de^	39.9 ^abcd^	29.1 ^abcd^	591.1 ^de^	699.3 ^abcd^	1451.0 ^d^	1638.4 ^ab^	2.6 ^ab^	2.5 ^ab^
Ethanol 50%	56.1 ^h^	80.2 ^efg^	120.4 ^de^	134.0 ^d^	41.8 ^abcd^	38.3 ^abcd^	481.1 ^e^	829.5 ^a^	1435.1 ^d^	1668.6 ^a^	1.7 ^b^	2.8 ^a^
Ethanol 70%	59.1 ^efg^	84.2 ^defg^	102.1 ^e^	132.3 ^d^	27.1 ^cd^	41.2 ^abcd^	604.3 ^de^	811.8 ^ab^	1438.3 ^d^	1646.3 ^a^	1.7 ^b^	3.0 ^a^
Ethanol 90%	68.4 ^efg^	65.7 ^efg^	131.9 ^de^	119.4 ^d^	26.1 ^abcd^	32.1 ^abcd^	661.2 ^abcd^	670.7 ^abcd^	1439.8 ^d^	1494.0 ^d^	3.0 ^a^	2.3 ^ab^
Water	52.0 ^g^	63.7 ^defg^	109.3 ^de^	110.9 ^de^	41.5 ^abcd^	23.8 ^d^	593.2 ^de^	655.6 ^abcd^	1422.4 ^d^	1487.6 ^d^	2.1 ^ab^	2.1 ^ab^

Gallic Acid Equivalents (GAE); Rutin Equivalent (RE); 2,2-diphenyl-1-picrylhydrazyl (DPPH); Trolox Equivalents (TE); Oxygen Radical Absorbance Capacity (ORAC); Ferric Reducing/Antioxidant Power (FRAP).

**Table 3:** *p*-Values found for the effects of conventional extraction parameters on the total polyphenol content, total flavonoid content, and antioxidant activity (DPPH, ORAC and FRAP) of extracts obtained from AR (*p* < 0.05 indicates significance).

Time	Factor	Total Polyphenols	Total Flavonoids	DPPH Inhibition %	ORAC	FRAP
2 h	Extraction Solvent	0.000	0.000	0.270	0.127	0.000
	Concentration	0.161	0.200	0.012	0.131	0.218
	Extraction Order	0.000	0.000	0.100	0.000	0.000
	Extraction Solvent Concentration	0.600	0.011	0.002	0.005	0.000
	Extraction Solvent × Extraction Order	0.641	0.095	0.001	0.000	0.000
	Concentration × Extraction Order	0.154	0.053	0.339	0.021	0.099
	Extraction Solvent × Concentration × Extraction Order	0.071	0.039	0.002	0.000	0.000
10 h	Concentration	0.000	0.000	0.000	0.040	0.662
	Extraction Order	0.000	0.000	0.298	0.613	0.101
	Concentration × Extraction Order	0.001	0.163	0.000	0.526	0.413
Comparing 2 h and 10 h	Extraction Time	0.000	0.000	0.015	0.000	0.002
	Concentration	0.279	0.000	0.000	0.610	0.000
	Extraction Order	0.000	0.000	0.160	0.000	0.000
	Extraction Time × Concentration	0.060	0.499	0.451	0.059	0.000
	Extraction Time × Extraction Order	0.005	0.000	0.014	0.000	0.000
	Concentration × Extraction Order	0.336	0.004	0.010	0.000	0.000
	Extraction Time × Concentration × Extraction Order	0.489	0.073	0.004	0.013	0.000

**Table 4:** Pearson’s Overall Correlation between the Antioxidant Assays in the 2-h Extraction (*p* < 0.05 indicates significance).

Extraction Time	Antioxidant Assay		Polyphenols	Flavonoids	DPPH	ORAC
2-h	Flavonoids	Pearson’s coefficient	0.322			
		*p*-value	0.000			
	DPPH Inhibition %	Pearson’s coefficient	0.110	0.269		
		*p*-value	0.228	0.003		
	ORAC	Pearson’s coefficient	0.201	0.199	0.162	
		*p*-value	0.025	0.028	0.076	
	FRAP	Pearson’s coefficient	0.455	0.338	0.084	0.450
		*p*-value	0.000	0.000	0.353	0.000
10-h	Flavonoids	Pearson’s coefficient	0.433			
		*p*-value	0.001			
	DPPH Inhibition %	Pearson’s coefficient	−0.165	−0.110		
		*p*-value	0.232	0.428		
	ORAC	Pearson’s coefficient	0.171	0.280	−0.161	
		*p*-value	0.217	0.041	0.244	
	FRAP	Pearson’s coefficient	0.003	0.109	−0.007	−0.075
		*p*-value	0.982	0.432	0.962	0.587
